# 

**Published:** 1890-06

**Authors:** 


### Communications.

### POST GRADUATE DENTAL ASSOCIATION OF THE UNITED STATES.

*Editor of the American Journal of Dental Science.*

The annual meeting of the Post Graduate Dental Association of the United States was held at Chicago, June 25, 1890, and the following gentlemen were elected officers:

President; Geo. H. Cushing, M. D., D.D.S., Chicago, Ill; Vice-President, Dr. R. H. Cool, Oakland, Cal; Secretary and Treasurer, Lewis S. Tenney, D.D.S., Chicago, Ill. Executive Committee, R. B. Tuller, D.D.S., Chicago, Ill; Dr. J. M. Gallehugh, Chenoa, Ill; Dr. G. W. Milton, Silvertown, Colo.

This association is but a year old, but it starts out with good prospects of becoming a large and popular national organization, and has a grand work before it. Its object aside from the same general one of most Dental Societies is to particularly encourage and stimulate post graduate studies and the establishment of facilities for the same in Dental Colleges. It also contemplates, when its membership will admit of it, establishing a systematic course of home study with benefits not unlike the Chatuaqua Literary Society perhaps, but the plan is not yet sufficiently developed to admit of outlining at this time.

While the name "Post Graduate" would imply an association of graduates only, the broad view is adopted of of extending the work among all legal practitioners who may desire to join and co-operate, but practitioners not graduates are not eligible to membership until they have past a post graduate or practitioners course in some reputable and recognized Dental College.

Members of the profession who desire to become members of the Post Graduate Association should correspond with the Secretary, Dr. Lewis S. Tenney, 96 State St., Chicago. The membership fee is $1. Annual dues, payable in advance $1. Certificates of membership are issued when the member duly qualifies. Membership may be obtained through correspondence when evidence of eligibility is presented.

---

### CHLORIDE OF METHYL SPRAY.

*Editor of the American Journal of Dental Science.*

DEAR SIR:—At a meeting of the Brooklyn Dental Society, held on Monday, March 24th, 1890, the following resolution was offered and adopted :

RESOLVED, That a copy of the last section of the report of the chairman of the Clinic Committee, relative to the chloride of methyl spray, be forwarded to each of the dental journals, with the request that they publish the same. In consideration of which, I herewith enclose a copy of said section, and sincerely trust you will give it space in your journal,

Very truly yours,

LOUIS SHAW, *Secretary.*

The following is a copy of the last section referred to:

" At a stated clinic of the Brooklyn Dental Society, held at 444 Fulton street, Brooklyn, on Monday March 24, 1890, Dr. M. L. Rhein repeated his clinic with chloride of methyl, and satisfied all present that he has suggested the most efficient and painless anæsthetic for sensitive dentine yet introduced. It is undoubtedly better than the spary from a nitrous-oxide cylinder, which was described and demonstrated recently before the Odontological Society, in New York. Your committee thinks this agent of so much consequence to us, and the method such a boon to suffering humanity, that it suggests that a copy of this part of the report be sent to all leading journals, that the matter may be brought to the attention of the profession in a prominent way, immediately.

"It has been previously demonstrated that dehydration produces anæsthesia in dentine, and that sprays have this effect. But all sprays, except this, produce considerable pain. The chloride of methyl spray acts so instantaneously that the pain is only a momentary shock at most, and is not complained of by the patients. It may seem that this is a strong report from your committee, but since the last clinic your committee has thoroughly investigated this method and seen it satisfactorily demonstrated in a large number of cases."

---

### CHICAGO DENTAL SOCIETY.

CHICAGO, May 13th 1890.

*Editor of the American Journal of Dental Science.*

At the annual meeting of the Chicago Dental Society, held on Tuesday, April 1st., 1890, the following officers were elected for the ensuing year: President, C. N. Johnson; First Vice-President, C. H. Thayer; Second Vice-President, I. A. Freeman; Secretary, A. E. Baldwin; Corresponding Secretary, T. L. Gilmer; Treasurer, E. D. Swain; Librarian, A. W. Harlan; Geo. H. Cushing, to succeed himself on the Executive Committee; C. F. Hartt, E. A. Royce, and S. B. Palmer, Board of Censors.

T. L. GILMER,

Corresponding Secretary.

### To Readers and Correspondents.

Communications intended for publication, and exchange journals and papers, should be addressed to the Editor of The American Journal of Dental Science, No. 845 N. Eutaw St. Baltimore, Md. All letters relating to the business of the Journal, or containing cash remittances, to be directed to Messrs. Snowden & Cowman. No. 9 W. Fayette Street, Baltimore, Md. Articles for publication should be received by the Editor at least two weeks previous to the first of the month on which the number in which it is designed for them to appear, is issued.

☞ The American Journal of Dental Science will contain fifty pages of reading matter in each number, making a volume of about Six Hundred pages,

EXCLUSIVE OF ADVERTISEMENTS.

